# A recall-rescue fuzzy layer over paired Z-DNA Hunter scans for fast Z-DNA detection

**DOI:** 10.1093/bib/bbag612

**Published:** 2026-11-09

**Authors:** Michal Petrovič, Oldřich Trenz, Filip Kledus, Václav Brázda, Jiří Šťastný

**Affiliations:** Department of Informatics, Mendel University in Brno, Zemědělská 1, 613 00 Brno, Czech Republic; Department of Informatics, Mendel University in Brno, Zemědělská 1, 613 00 Brno, Czech Republic; Institute of Biophysics, Czech Academy of Sciences, Královopolská 135, 612 00 Brno, Czech Republic; National Centre for Biomolecular Research, Faculty of Science, Masaryk University, Kamenice 5, 625 00 Brno, Czech Republic; Institute of Biophysics, Czech Academy of Sciences, Královopolská 135, 612 00 Brno, Czech Republic; Department of Informatics, Mendel University in Brno, Zemědělská 1, 613 00 Brno, Czech Republic

**Keywords:** Z-DNA, bioinformatics, sequence analysis, candidate prioritization

## Abstract

Accurate detection of Z-DNA-forming regions is limited by the trade-off between sensitivity and specificity in sequence-based methods. We present Z-DNA-Fuzzy Hunter 1.1.0—a fast and interpretable protocol that extends Z-DNA Hunter through a dual-scan strategy combined with a fuzzy expert layer. A permissive scan (model 2, minimum length 6 bp, threshold 30%) captures a broad candidate set, while a stringent scan (model 2, minimum length 10 bp, threshold 60%) provides high-confidence support. These outputs are integrated using fuzzy inference incorporating sequence signal, cross-scan support, genomic context, and bias penalties. On the audited 385-locus Shin analysis, the protocol achieves balanced accuracy 0.883 (recall 0.862, specificity 0.904), comparable with the published Z-DNABERT operating point, while alternative modes increase specificity to 0.973. The paired scans are combined by a transparent, configurable prioritization layer with an adjustable recall–specificity trade-off. The local workflow requires only a FASTA (Fast-All sequence alignment format), sequence and transcription start site annotation. The tool is freely available at https://github.com/mifo123/zdna-fuzzy-hunter.

## Introduction

Z-DNA is a non-B DNA structure characterized by a left-handed double helix with zigzag sugar-phosphate backbone, usually formed by alternating purine and pyrimidine sequences. Since its discovery and structural characterization between the late 1960s and early 1980s (reviewed by [1]), Z-DNA has received increasing attention thanks to the growing evidence of its potential biological functions. Although intrinsically unstable under physiological conditions, Z-DNA can be stabilized by negative supercoiling and specifically recognized by Z-DNA-binding proteins (ZBPs), which promote its formation and persistence *in vivo* [2–4]. Multiple studies have now confirmed that Z-DNA is not just a mere topological curiosity, but a biologically relevant structure, associated with transcriptionally active regions and regulatory genomic elements, making its reliable identification important for understanding genome function [5–8]. Well-known proto-oncogene MYC proto-oncogene has been shown to recruit chromatin remodeling complex Facilitates Chromatin Transcription (FACT) that facilitates formation of Z-DNA, which further promotes activation of transcription after the loading of RNA Polymerase II [9, 10]. Another recent work also demonstrates that Z-DNA formation is dynamically linked to higher-order genome organization and regulatory processes, including chromatin compartmentalization and cell state transitions [11].

On the other hand, Z-DNA sequences have also been connected to genomic instability, stalling of transcription, DNA breaks, and somatic mutations, often associated with cancer. The prevention of Z-DNA formation by the ZBTB43 zinc finger protein has been confirmed in mouse germ cells as a mechanism that maintains genome stability [12]. Mitochondrial damage, also linked with many diseases, can lead to release of mitochondrial DNA into the cytosol, where Z-DNA-prone regions may subsequently be stabilized by ZBP1. The B to Z transition is thus one of the indicators of mitochondrial genome instability, which can further lead to activation of innate immune signaling, autoimmunity, and inflammation [13, 14].

Computational detection of Z-DNA-forming sequences spans several algorithmic families. Z-Hunt II estimates thermodynamic Z-DNA propensity and established genome-scale mapping of potential Z-DNA-forming sequences [15]. Z-DNA Hunter [16] implements explicit sequence scoring related to the alternating purine–pyrimidine logic available through the non-B DNA Motif Search Tool [17], while ZSeeker [18] provides a recent machine-learning sequence classifier. Z-DNA GENome Identification Engine (Z-GENIE) wraps Z-Hunt in an R/Shiny interface with interactive visualization and local or hosted workflows [19]. Deep-learning approaches include DeepZ, a deep neural network integrating sequence and omics features [20], and Z-DNABERT, a transformer derived from DNABERT [21, 22]. These approaches differ in input requirements, execution cost, and interpretability [23]; no single benchmark row establishes universal superiority across assays and genomes.

To address the single-threshold recall–specificity trade-off, we developed Z-DNA-Fuzzy Hunter, a dual-configuration Z-DNA Hunter workflow with an interpretable fuzzy integration layer (Fig. 1). The method is intended as a tunable prioritization system, not a universal replacement for Z-Hunt II, Z-GENIE, ZSeeker, DeepZ, or Z-DNABERT. Its advantages are local Central Processing Unit (CPU) execution, explicit rules, and adjustable operating points; its costs are additional parameters, dependence on the upstream feature semantics and the need to recalibrate thresholds when species, assays, or class prevalence change.

**Figure 1:**
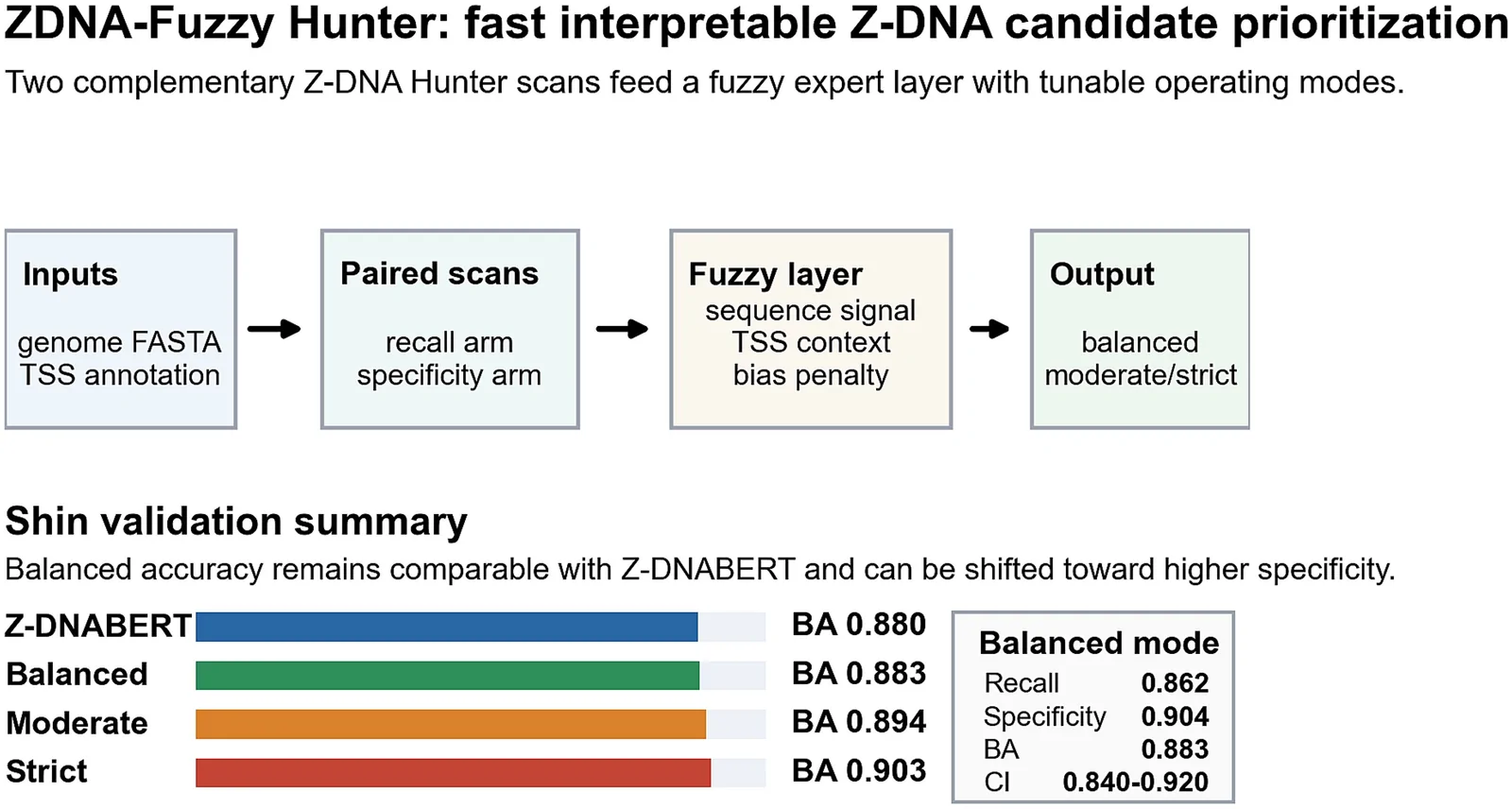
Overview of Z-DNA-Fuzzy Hunter and the main Shin validation results; genome FASTA and TSS annotation are processed by permissive and stringent Z-DNA Hunter scans and integrated by the fuzzy layer; BA denotes balanced accuracy; the balanced-mode 95% confidence interval (CI; 0.840–0.920) is the percentile interval from 2000 nonparametric bootstrap resamples of the 385 labeled locus–prediction pairs using random seed 20260604.

## Materials and methods

### Study design and validation data

The public HG Shin source cohort comprised 316 experimentally positive and 75 experimentally negative loci reported by Shin *et al*. [7] in the NCBI36/hg18, the human reference genome assembly Build 36 (NCBI36/hg18) coordinate system. The analysis was restricted to the 24 primary chromosomes (chr1–chr22, chrX, and chrY); six *_random-contig records were excluded, leaving 312 positive and 73 negative loci. Z-DNABERT was not used as an input feature and appears only as a published aggregate comparison. Nearest-transcription start site (TSS) distance was calculated from 21 719 Ensembl release 52 TSS records in Hum_ENSEMBL52.sga, obtained through the Mammalian Gene Collection / Eukaryotic Promoter Database (MGA/EPD) Ensembl 52 collection from BioMart for the March 2006 NCBI36 assembly [24]. Sequence Genome Annotation (SGA) point positions were interpreted as one-based and converted internally to zero-based coordinates. Performance measures were recall, specificity, precision, F1 score, Matthews correlation coefficient (MCC), balanced accuracy (BA), and Youden’s J (recall + specificity − 1) [25]. The balanced-accuracy confidence interval was computed by 2000 percentile bootstrap resamples of labeled locus–prediction pairs with random seed 20260604.

For context-dependence checks, positive, and negative loci were summarized separately by nearest-TSS distance, and outcome groups were compared using both means and medians. Per-locus scores, raw Hunter features, predictions for all operating modes and deterministic ranks are reported in Supplementary Table S1.

### Upstream Z-DNA Hunter scans

The Shin preset uses two model 2 Z-DNA Hunter scans: m2_l6_t30 (minimum motif length 6 bp, threshold 30%) for recall rescue and m2_l10_t60 (minimum motif length 10 bp, threshold 60%) for confirmation. Both use pair scores guanine-cytosine (GC) = 2, guanine-thymine/adenine-cytosine (GT/AC) = 1, adenine-thymine (AT) = 0.5, and all other pairs = 0. The public FASTA headers are interpreted as one-based closed intervals and converted to zero-based half-open coordinates before both scans are rebuilt locally. Hits are represented as half-open (start, end) intervals, merged when overlapping, and summarized by hit count, weighted hit fraction, strict-hit fraction, maximum raw score, best interval length, and overlap. The original Application Programming Interface (API) export uses inclusive end coordinates and is converted to half-open intervals before merging.

### Parameter selection and internal validation

Candidate scan pairs were screened on the Shin data. Fuzzy coefficients were selected from 3500 deterministic random parameter vectors (seed 20260604) using five stratified folds. For a given vector, each held-out fold used a threshold selected on the other four-folds; however, aggregate performance across those same folds selected the final coefficient vector. Consequently, the reported five-fold result is tuning-aware and potentially optimistic, not fully nested validation. The final threshold 19.157 and all coefficients were then fixed for the all-Shin operating point. For both independent checks, the entire Shin-derived model—scan settings, fuzzy coefficients, membership functions, rule base, and balanced threshold—was frozen before the experimental data were evaluated.

### Independent U2OS Z-DNA ChIP-seq check

The frozen Shin-derived model was evaluated on the independent human U2OS Z-DNA chromatin immunoprecipitation followed by high-throughput sequencing' (ChIP-seq) dataset series GSE290662. We used the two wild-type Z22-IP tracks GSM8818990 and GSM8818991 and their matched input tracks GSM8818982 and GSM8818983, all aligned by the data submitters to hg19. Chromosome 22 was scanned with the unchanged Shin-publication preset, balanced threshold 19.157, and GENCODE v19 transcript TSS annotation [26], producing 290 220 merged candidates. A deterministic sample of 2000 candidates from each fuzzy-score decile (20 000 total; seed 20260803) was analyzed. Exact mean BigWig signal was measured in a 500-bp window centered on each candidate. A window was defined a priori as meeting the replicate-consistent high-signal criterion when Z22-IP exceeded its matched input in both replicates and mean Z22-IP was in the top 10% of sampled windows. We report per-decile rates, the highest-versus-lowest-decile risk ratio, and a percentile 95% confidence interval for the rate difference from 2000 bootstrap resamples (seed 20260804). Spearman score-to-signal correlation and a >20-kb-from-TSS sensitivity analysis were also calculated. Supplementary Table S5 and Supplementary Data S6 provide aggregate and sampled per-candidate outputs.

### Independent rice Z-DNA ImmunoPrecipitation followed by sequencing (ZIP-seq) check

The same frozen Shin-derived model was additionally evaluated using the original data associated with doi:10.1111/pbi.14585. GEO series GSE252954 provides Nipponbare rice leaf ZIP-seq Z22-IP replicates GSM8010917/GSM8010918, input GSM8010919, immunoglobulin G (IgG) GSM8010920, and CUT&Tag replicates GSM8010921/GSM8010922 [27]. The unchanged Shin preset and threshold were applied to Michigan State University/University of Georgia (MSU/UGA) Rice Genome Annotation Release 7 chromosome 1 [28], producing 558 171 merged candidates. We again sampled 2000 candidates per fuzzy decile (20 000 total; seed 20260805) and measured exact mean signal in centered 500-bp windows. The prespecified primary high-signal criterion required both ZIP-seq immunoprecipitation (IP) replicates to exceed both input and IgG and mean IP to be in the top 10% of sampled windows. We report extreme-decile rates, risk ratio, a 2000-resample bootstrap confidence interval for their difference, continuous ZIP-seq enrichment correlations, and CUT&Tag signal correlations. Supplementary Table S7 and Supplementary Data S8 provide aggregate and candidate-level outputs.

### Fuzzy expert layer

All inputs and derived variables are scaled to 0–100. Four weighted composites are calculated—sequence signal (S), context (C), evidence (E), and bias (B)—together with two auxiliary variables passed to the rules: validation feasibility (F) and cross-model balance (Q) (Table 1). Thus, the implementation consistently uses six component-level variables, of which four are weighted composites. The five linguistic sets for S, C, E, F, and Q are trapezoids [a,b,c,d]: very low [0,0,8,22], low [12,24,36,50], medium [38,50,62,74], high [62,74,84,94], and very high [86,94,100,100]. Bias uses very low [0,0,6,16], low [8,18,28,40], medium [30,42,55,68], high [58,70,82,92], and very high [84,94,100,100]. Membership is 1 on [b,c], linear on [a,b] and [c,d], and 0 outside [a,d]. This construction follows established principles for explainable rule-based and heuristically designed fuzzy inference systems [29, 30].

**Table 1:** Complete Takagi–Sugeno rule base; all 12 rules are evaluated in balanced, moderate, and strict modes; the modes differ only through the post-threshold filters described below.

Rule	Antecedent	Singleton
AF1	S very high AND E very high AND B very low	98
AF2	S very high AND E high/very high AND B very low/low	93
AF3	S high AND E very high AND C medium or higher AND B very low/low	89
AF4	S high AND E high AND B low/medium	82
AF5	S medium AND E high/very high AND C high	72
AF6	S high AND E medium AND C low	64
AF7	S medium AND E medium AND B low	58
AF8	S very low AND E low	12
AF9	B high/very high AND E low/medium	22
AF10	S high AND Q low	48
AF11	S very high AND Q very high AND F medium	91
AF12	S low AND B high/very high	14

A slash denotes logical OR. S, E, C, and B denote signal, evidence, context, and bias; Q and F denote cross-model balance and feasibility. Supplementary File S2 provides the identical machine-readable model specification.

The fixed composites are S = clamp(0.379210 W + 0.051894Z + 0.198225H + 0.191368 M1 + 0.179302 M2 + 3.089634A/100), C = clamp(0.476750 T + 0.021460P + 0.408899 K), E = clamp(0.459564R + 0.130052 M1 + 0.355236 M2 + 0.055148Q + 5.240791A/100), and B = clamp(0.296978D + 0.162730 U + 0.284105O + 0.256187 N). W is weighted Hunter support, Z maximum Hunter score, H scan vote, M1/M2 model-family support, A strict/all-scan support, T TSS proximity, P promoter indicator, K context-support bin, R source support, D repeat signal, U disagreement, O model 2-only support, and N no-hit penalty. Clamp restricts values to [0,100]. The base score is clamp(4.364880 + 0.439786S + 0.448789E + 0.010134C + 0.101291F − 0.471126B). Rule antecedents use minimum for AND and maximum for OR; activated singleton consequents are averaged by firing strength. The final score is clamp(0.850764 × base+0.149236 × rule score). Therefore, the rule system contributes 14.9%, while the weighted component path contributes 85.1%.

### Operating points

All modes first require at least one upstream grid hit and a fuzzy score ≥19.157. Balanced mode applies no additional filter. Moderate mode excludes a candidate only when TSS distance ≤10 bp and score ≤19.5. Strict mode excludes a candidate only when all three conditions hold: TSS distance ≤50 bp, signal ≤38.51, and maximum Hunter overlap ≤2.0%. These post-threshold filters were selected on Shin data and should not be assumed optimal elsewhere.

### Implementation and reproducibility

Z-DNA-Fuzzy Hunter 1.1.0 is implemented in Python 3.10 or later under Massachusetts Institute of Technology License (MIT). The default backend is a fully local, Java-compatible Z-DNA Hunter implementation; the Institute of Biophysics of the Czech Academy of Sciences (IBP) API remains optional. The local and archived API pipelines produced identical coordinates and calls for 33 125 selected dm6 candidates, with only 16 scores differing by 0.0001 because archived API raw scores were rounded. Inputs are FASTA plus TSS annotation in SGA, BED (Browser Extensible Data), or delimited text, with optional BED-like context tracks; output is CSV (comma-separated values) or bedGraph. All genomic outputs are zero-based and half-open. SGA is treated as one-based by automatic detection, while negative-strand BED TSS positions use end −1. Model components are exposed as functions with docstrings. Unit tests cover coordinate normalization, local hit coordinates, and complete model export. Dependencies, Python version, command examples, and the coordinate convention are documented in the README.

### Runtime benchmarking

Wall-clock time was measured on a MacBook Air with an Apple M2 CPU (8 cores), 16 GB memory, macOS 26.6, and Python 3.12.13 without network access. The end-to-end test scanned the eight principal dm6 chromosomes with the genome-balanced preset (m2_l8_t30/m2_l10_t60), strict mode and minimum score 50, merged 482 286 components, and wrote 33 125 calls. Feature-table scoring was repeated five times. Stage timings are reported in Table 6 and Supplementary Table S4.

## Results and discussion

On the 385 Shin loci, raw m2_l6_t30 recovered nearly all positives (recall 0.987) but classified most negatives as positive (specificity 0.082), demonstrating why it is a recall-rescue arm rather than a standalone classifier (Table 2). The paired balanced model yielded 269 true positives, 66 true negatives, 7 false positives, and 43 false negatives, corresponding to recall 0.862, specificity 0.904, precision 0.975, F1 score 0.915, MCC 0.667, and BA 0.883. Moderate and strict modes raised specificity to 0.945 and 0.973 with recall 0.843 and 0.833, respectively. Five-fold internal evaluation gave recall 0.856, specificity 0.904, and BA 0.880, but this is a tuning-aware estimate because the same folds selected the coefficient vector.

**Table 2:** Recall-rescue configuration screening on Shin loci.

Configuration/operating point	Recall	Specificity	BA	Comment
m2_l6_t30, raw overlap	0.987	0.082	0.535	High recall, too noisy alone
m2_l6_t30 + m2_l10_t60, five-fold internal CV	0.856	0.904	0.880	Selected model; tuning-aware, not nested
m2_l6_t30 + m2_l10_t60, all Shin	0.862	0.904	0.883	Balanced threshold
m2_l6_t12 + m2_l10_t60, all Shin	0.865	0.890	0.878	Higher recall, lower BA
m2_l8_t12 + m2_l10_t60, high-specificity screening pair	0.779	0.973	0.876	High-specificity baseline
m2_l6_t30 + m2_l10_t60, recall-targeted	0.885	0.822	0.853	Recall match, specificity loss

CV denotes the tuning-aware five-fold internal evaluation; it is not nested cross-validation.

Configuration screening (Table 2) selected m2_l6_t30 plus m2_l10_t60 because the permissive arm provided candidate recall and the stringent arm supplied confirmation. The all-Shin threshold is an apparent operating-point result, while the cross-validated row is an internal, tuning-aware estimate. These results should not be interpreted as independent evidence against overfitting.

The balanced operating point is comparable to the published Z-DNABERT hg18 threshold-0.25 row (recall 0.877, specificity 0.890, and BA 0.880); the small numerical difference does not establish superiority because the values were not produced in one independently locked evaluation. Table 3 also includes the raw Z-DNA Hunter baselines. Z-Hunt II, Z-GENIE, ZSeeker, and DeepZ are discussed by method family in Supplementary Table S3 but were not rerun per locus; reporting invented cross-tool numbers would confound versions, inputs, and coordinate processing. Supplementary Table S1 enables direct per-locus comparison and re-ranking between the fuzzy score and raw Hunter evidence. For the balanced operating point, the confusion matrix comprised 269 true positives, 66 true negatives, 43 false negatives, and 7 false positives, confirming that the achieved BA reflects both high sensitivity and strong discrimination of experimentally negative loci (Table 4).

**Table 3:** Main Shin benchmark comparison; Z-DNABERT and published Z-DNA Hunter rows are external aggregate comparators; the fuzzy rows use the Shin implementation described here.

Model/operating point	Recall	Specificity	BA	Youden J
Final fuzzy pair, balanced threshold	0.862	0.904	0.883	0.766
Final fuzzy pair, moderate specificity mode	0.843	0.945	0.894	0.788
Final fuzzy pair, strict specificity mode	0.833	0.973	0.903	0.806
Z-DNABERT hg18 threshold 0.25, published	0.877	0.890	0.880	0.770
Final fuzzy pair, five-fold internal CV	0.856	0.904	0.880	0.760
Z-DNA Hunter model 2 size 10, published	0.726	0.973	0.850	0.700
Z-DNA Hunter model 2 size 8 threshold 12%, published	0.877	0.740	0.810	0.620

**Table 4:** Confusion matrix for the balanced fuzzy operating point on all Shin loci at threshold 19.157.

	Predicted positive	Predicted negative
Experimentally positive	269	43
Experimentally negative	7	66

Post hoc ablation shows the largest balanced-accuracy losses after removal of Hunter sequence signal (−0.092) or evidence/confirmation support (−0.074). Context removal reduced BA by 0.021 and TSS removal by 0.017. The model is therefore driven mainly by upstream sequence evidence, although TSS context can alter borderline calls. Figure 2 visualizes the losses and Table 5 provides exact values.

**Figure 2:**
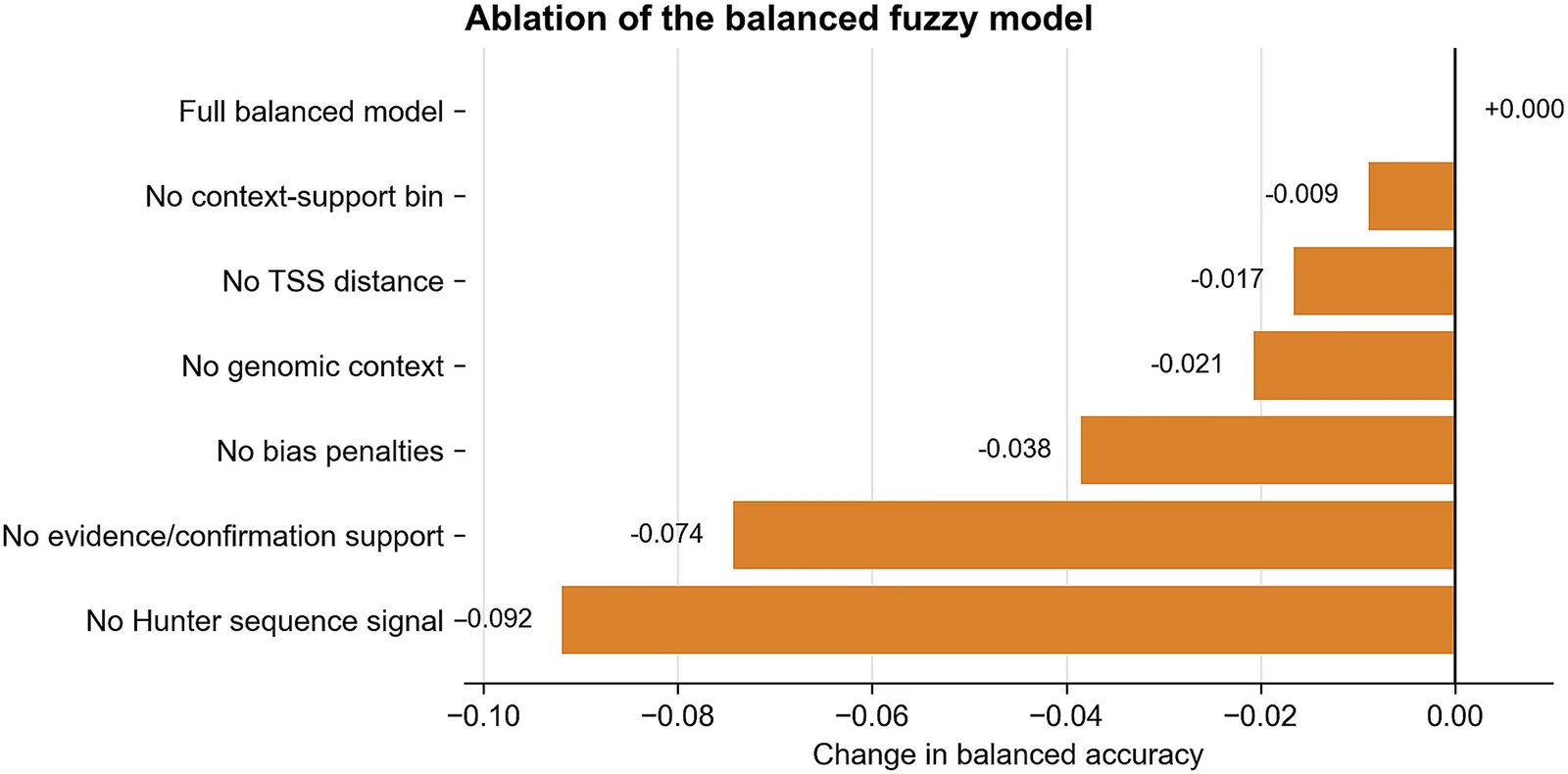
Ablation analysis of the balanced fuzzy operating point; bars show the change in BA after removing each input group; larger negative values indicate greater contribution. Horizontal bars show balanced-accuracy change after input removal. The largest losses occur without Hunter sequence signal (−0.092) and without evidence support (−0.074); other losses range from −0.038 to −0.009.

**Table 5:** *Post hoc* ablation analysis for the balanced operating point.

Model variant	Recall	Specificity	BA	Delta BA
Full balanced model	0.862	0.904	0.883	0.000
No Hunter sequence signal	0.596	0.986	0.791	−0.092
No evidence/confirmation support	0.631	0.986	0.809	−0.074
No bias penalties	0.785	0.904	0.845	−0.038
No genomic context	0.766	0.959	0.862	−0.021
No TSS distance	0.747	0.986	0.867	−0.017
No context-support bin	0.872	0.877	0.874	−0.009

Delta BA is relative to the full model.

**Table 6:** Local runtime benchmark.

Stage	Time (s)	Scope
FASTA/TSS loading	1.875	Eight principal dm6 chromosomes
Two local Hunter scans	77.457	Genome-balanced: m2_l8_t30 and m2_l10_t60
Merge + fuzzy scoring	8.975	482 286 candidate components
CSV output	0.470	Strict mode; minimum score 50; 33 125 calls
End-to-end wall time	89.580	Apple M2 laptop; no network
Precomputed feature-table scoring	0.910	Median of five runs; 0.900–0.960 s

Stage times are instrumented values; wall time is the external end-to-end measurement.

Nearest-TSS distributions differed between classes but overlapped substantially. Positive loci had mean distance 58 934 bp and median 3320 bp (interquartile range 187–44 475); negative loci had mean 51 547 bp and median 12 656 bp (interquartile range 294–31 652). A total of 125/312 positives (40.1%) and 26/73 negatives (35.6%) were within 1 kb of a TSS, including 26 positive and 11 negative TSS-overlapping loci. All seven balanced-mode false positives were within 1 kb, indicating that TSS proximity can lift borderline candidates but is not by itself class-specific. Figure 3 shows the representative locus and outcome-stratified distances.

**Figure 3:**
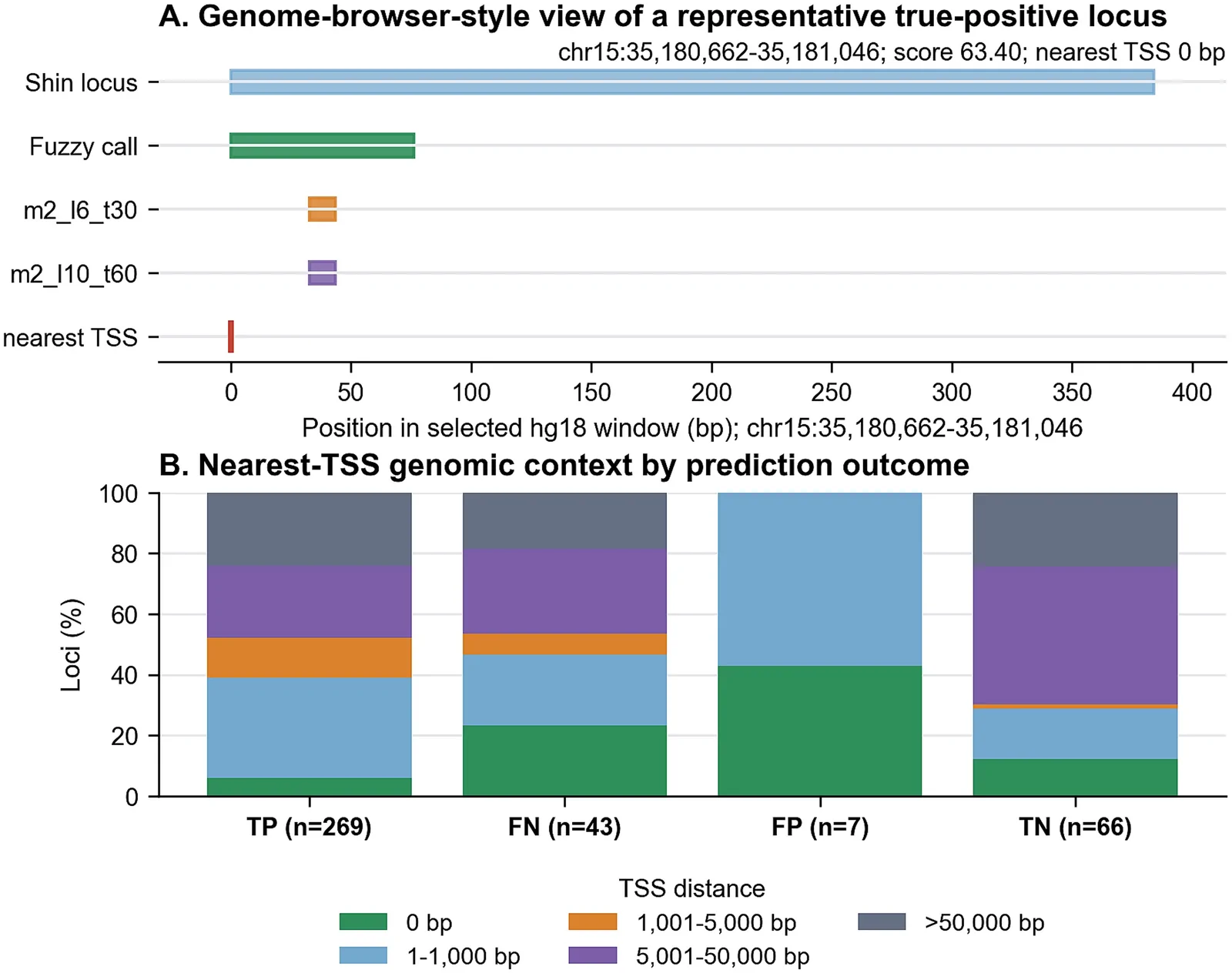
Genomic-context inspection of the balanced Shin validation; panel A shows one true-positive locus with the fuzzy and paired Hunter calls; panel B stratifies true-positive (TP; n = 269), false-negative (FN; n = 43), false-positive (FP; n = 7), and true-negative (TN; n = 66) outcomes by nearest-TSS distance; distance bins are 0 bp, 1–1000 bp, 1001–5000 bp, 5001–50 000 bp, and >50 000 bp.

The fuzzy score was informative as a ranking: the top 10%, 25%, and 50% had precision 1.000, 0.990, and 0.995, with positive recall 0.125, 0.308, and 0.615. On the full Shin development dataset, the apparent receiver operating characteristic curve / area under the curve (ROC AUC) was 0.926 and the apparent average precision was 0.980 against prevalence 0.810 (Fig. 4). Across all loci, the Spearman correlation of fuzzy rank was 0.877 with weighted Hunter-hit fraction, 0.753 with maximum Hunter overlap, and 0.430 with maximum raw Hunter score. The incomplete correlations quantify reordering introduced by context, evidence, bias, feasibility, and the rules; every rank shift is available in Supplementary Table S1.

**Figure 4:**
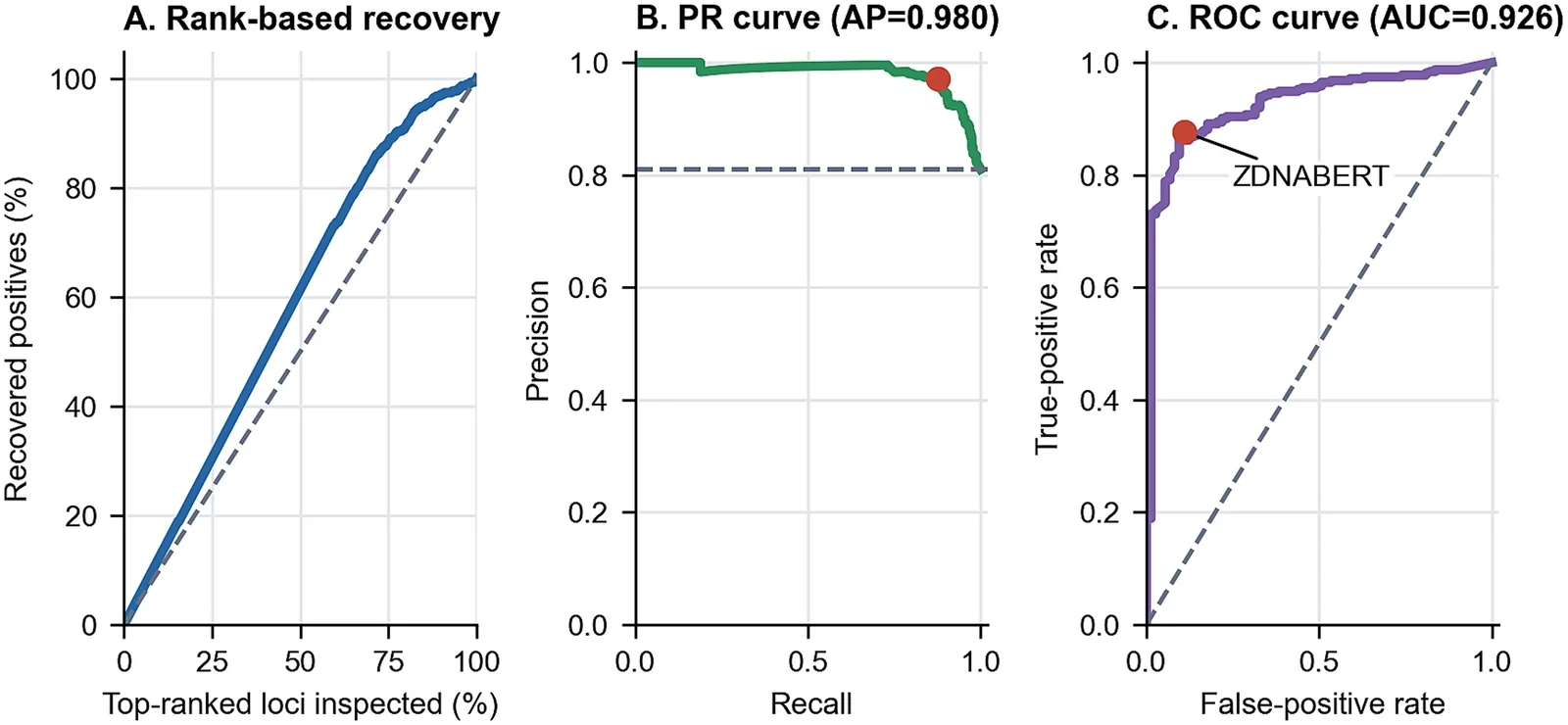
Rank-based and threshold-independent evaluation on the Shin benchmark; panel A shows positive-locus recovery as candidates are inspected by fuzzy rank; panels B and C show precision-recall and receiver operating characteristic (ROC) curves. The published Z-DNABERT hg18 threshold-0.25 operating point is indicated for comparison in panels B and C. Three panels show cumulative recovery by fuzzy rank, a precision-recall curve with average precision 0.980, and a receiver operating characteristic curve with an area under the curve (AUC) of 0.926.

Residual errors were sequence-evidence limited. True positives had mean/median maximum Hunter overlap 10.4%/8.9%, mean/median fuzzy score 49.5/62.1, and strict-support fraction 0.725. False negatives had mean/median overlap 1.9%/2.1%, mean/median fuzzy score 16.9/18.8, and no strict support. False positives had mean/median overlap 2.0%/1.8%, mean/median score 25.6/19.5, and mean/median TSS distance 91.6/3 bp. True negatives had mean/median overlap 1.8%/1.8%, mean/median score 16.5/18.4, and mean/median TSS distance 57 004/16 605 bp. These summaries show why both means and medians are needed for skewed distance distributions.

The results support a two-stage interpretation: permissive scanning broadens the candidate pool, and a stringent scan plus fuzzy scoring orders or filters that pool. The fuzzy layer can ingest a precomputed feature table, but substituting Z-Hunt II, ZSeeker, or another detector is not plug-and-play: source-specific scores must be normalized to the documented features and the fuzzy coefficients and thresholds must be independently recalibrated. This modularity is therefore architectural, not evidence that the current weights generalize to arbitrary baseline tools.

### Independent U2OS signal enrichment

The frozen Shin-derived model provided limited but measurable enrichment in the independent U2OS analysis. Of the 2000 sampled candidates in each extreme decile, 201/2000 (10.1%) in the highest fuzzy-score decile and 148/2000 (7.4%) in the lowest decile met the replicate-consistent high-Z22-IP definition, giving a risk ratio of 1.36 and a rate difference of 0.027 (bootstrap 95% CI 0.009–0.044). The decile pattern was not monotonic, however, and the Spearman correlation between continuous fuzzy score and mean log2 Z22-IP/input signal was approximately zero; the corresponding raw Hunter-score correlation was −0.013. Among candidates ˃20 kb from a TSS, positive rates were 6/125 (4.8%) in the highest decile, and 66/1071 (6.2%) in the lowest. Thus, the high-score tail was modestly enriched globally, but the absence of continuous or distal support indicates that the effect is largely TSS-associated. This independent check does not establish a calibrated second-dataset classifier, whole-genome peak sensitivity, or cross-species transfer.

### Independent rice ZIP-seq enrichment

The independent original-data check using the dataset associated with doi:https://doi.org/10.1111/pbi.14585 showed stronger extreme-decile separation on rice chromosome 1. The prespecified ZIP-seq-positive criterion was met by 336/2000 (16.8%) highest-decile and 56/2000 (2.8%) lowest-decile candidates, giving a risk ratio of 6.0 and a rate difference of 0.140 (bootstrap 95% CI 0.121–0.158). The full decile pattern was non-monotonic (21.0% in decile 7 and 1.7% in decile 8). Continuous fuzzy score correlated modestly with ZIP-seq enrichment (Spearman 0.162) and weakly with mean CUT&Tag signal (0.065); corresponding raw Hunter correlations were 0.276 and 0.106. ZIP-seq and CUT&Tag replicate Pearson correlations were 0.890 and 0.911. However, no highest-decile sampled candidate was ˃20 kb from a TSS, so a distal sensitivity analysis was impossible. This cross-species result supports enrichment in the model’s TSS-proximal high-score tail, but cannot separate transferable sequence recognition from the model’s explicit TSS component or establish whole-genome peak sensitivity.

### Algorithmic trade-offs and runtime profile

The tool families serve different purposes. Z-Hunt II uses thermodynamic propensity [15]; Z-GENIE improves access to Z-Hunt with an R/Shiny workflow and reports that large-genome Z-Hunt scans can take up to ~2 h depending on input [19]; Z-DNA Hunter provides an explicit sequence-scoring baseline [16]; ZSeeker is a recent machine-learning sequence method [18]; DeepZ is a deep neural network rather than a transformer [20]; and Z-DNABERT is transformer-based [21, 22]. Supplementary Table S3 distinguishes quantitative rows rerun here from qualitative tool-scope comparisons.

The local *Drosophila melanogaster* dm6 benchmark separated scanning from post-processing. FASTA/TSS loading took 1.875 s, two Hunter scans 77.457 s, merging and fuzzy scoring 8.975 s, and CSV writing 0.470 s; total measured time before summary output was 88.776 s and external wall time 89.580 s. Fuzzy scoring plus CSV output on the precomputed 33 125-row feature table took a median 0.910 s across five runs (range 0.900–0.960 s). The dominant cost is therefore upstream scanning, not the fuzzy arithmetic.

Z-DNA-Fuzzy Hunter is most useful when transparent local prioritization and an adjustable call set matter. Its limitations are explicit: a second scan adds runtime, the coefficient count increases overfitting risk, TSS-associated features can bias performance when class-specific TSS distributions differ, and the Shin internal validation is not nested. The GSE290662 analysis was restricted to hg19 chromosome 22, one cell line, a deterministic sample, and a fixed-window signal criterion; its continuous and distal results were null. The original-data GSE252954 result provides cross-species evidence on rice chromosome 1, but every highest-decile sampled candidate was within 20 kb of a TSS and the CUT&Tag correlation was weak. It therefore cannot isolate species transfer from TSS-driven ranking or establish whole-genome peak sensitivity. Independent evaluation on held-out peak calls, additional chromosomes, and the mouse GSE224553 dataset remains required.

## Conclusion

Z-DNA-Fuzzy Hunter 1.1.0 provides a local and interpretable strategy for ranking Z-DNA candidates. On Shin loci, its balanced operating point was comparable to the published Z-DNABERT row, but the internal cross-validation is tuning-aware and does not support a superiority claim. Frozen-model U2OS and original-data rice checks both showed highest-decile enrichment. U2OS continuous and distal analyses were null, whereas rice ZIP-seq correlation was modest and all highest-decile rice candidates were TSS-proximal. The evidence supports limited TSS-associated prioritization, including a constrained cross-species signal, but does not demonstrate broad transfer. The complete model, per-locus outputs, independent-check outputs, tests, coordinate conventions, MIT license, and local backend make these assumptions auditable.

**Key Points**

- Z-DNA-Fuzzy Hunter combines permissive and stringent local sequence scans to recover and prioritize candidate Z-DNA-forming regions.
- A fixed fuzzy rule base integrates scan agreement, sequence strength, transcription start site (TSS) context, and bias indicators into an interpretable score.
- The audited 385-locus Shin analysis yielded balanced accuracy 0.883, with alternative operating modes for higher specificity.
- Independent experiments checks detected enrichment in the high-score tail and showed that the current model remains strongly TSS-dependent.
- The local Python implementation exposes model parameters and provides auditable per-locus scores for downstream analysis.

## Supplementary Material

Supplementary_Materials_bbag612

## Data Availability

Source code (Z-DNA-Fuzzy Hunter 1.1.0), the MIT license, tests, and released inputs are available at https://github.com/mifo123/zdna-fuzzy-hunter. The versioned release, including the tool and supplementary files, is preserved on Zenodo (DOI: 10.5281/zenodo.22279840). The Shin analysis is reconstructed from the public HG Shin FASTA cohort distributed with Z-DNABERT: 391 source records (316 positive and 75 negative), with six *_random-contig records excluded to give the 385 primary-chromosome analysis loci (312 positive and 73 negative). The Shin TSS file is the Ensembl 52/MGA collection Hum_ENSEMBL52.sga for NCBI36/hg18 (https://epd.expasy.org/mga/hg18/ensembl52/ensembl52.html). The independent U2OS check used original GEO series GSE290662 with hg19/GENCODE v19. The rice check used the original data associated with doi:https://doi.org/10.1111/pbi.14585, GEO series GSE252954, and MSU/UGA Release 7.
